# Correlation of extracellular polymeric substances and microbial community structure in denitrification biofilm exposed to adverse conditions

**DOI:** 10.1111/1751-7915.13633

**Published:** 2020-07-23

**Authors:** Shuo Wang, Liling Zhi, Wei Shan, Hui Lu, Qiao Xu, Ji Li

**Affiliations:** ^1^ Jiangsu Key Laboratory of Anaerobic Biotechnology School of Environment and Civil Engineering Jiangnan University Wuxi 214122 China; ^2^ Jiangsu Engineering Laboratory for Biomass Energy and Carbon Reduction Technology Jiangnan University Wuxi 214122 China; ^3^ Jiangsu College of Water Treatment Technology and Material Collaborative Innovation Center Suzhou 215009 China; ^4^ Department of Civil Engineering Schulich School of Engineering University of Calgary Calgary T2N 1N4 Canada; ^5^ School of Environmental Science and Engineering Sun Yat‐sen University Guangzhou 510006 China

## Abstract

Microbial community may respond to different adverse conditions and result in the variation of extracellular polymeric substances (EPS) in denitrification biofilm; this study discovered the role of EPS in accordance with the analysis of cyclic diguanylate (c‐di‐GMP) and electron equilibrium (EE) under low organic loading rate, shock organic loading rate and low temperature conditions. Good nitrate removal performance could be achieved under shock organic loading rate and low temperature conditions; however, owing to the low organic loading rate, the carbon source was preferentially utilized for biomass growth. Tightly bound EPS (TB‐EPS) contents progressively increased and facilitated cell adhesion and biofilm formation. The stable TB protein (TB‐PN) content in TB‐EPS built a cross‐linked network to maintain internal biofilm structure and led to the rapid biosynthesis of polysaccharides, which could further enhance microbial adhesion and improve nitrate removal. C‐di‐GMP played an important role in biomass retention and biofilm formation, based on the correlation analysis of c‐di‐GMP and EPS. TB polysaccharide (TB‐PS) contents presented a significant positive correlation with c‐di‐GMP content, microbial adhesion and biofilm stabilization was further enhanced through c‐di‐GMP regulation. In addition, a remarkable negative correlation between electron deletion rate (EDR) and TB‐PN and TB‐PS was discovered, and TB‐PS was required to serve as energy source to enhance denitrification according to EE analysis. Surprisingly, dynamic microbial community was observed due to the drastic community succession under low temperature conditions, and the discrepancy between the dominant species for denitrification was found under shock organic loading rate and low temperature conditions. The notable increase in bacterial strains *Simlicispira*, *Pseudomonas* and *Chryseobacterium* was conducive to biofilm formation and denitrification under shock organic loading rate, while *Dechloromonas* and *Zoogloea* dramatically enriched for nitrate removal under low temperature conditions. The high abundance of *Dechloromonas* improved the secretion of EPS through the downstream signal transduction, and the c‐di‐GMP conserved in *Pseudomonas* concurrently facilitated to enhance exopolysaccharide production to shock organic loading rate and low temperature conditions.

## Graphical abstract

The discrepancy of c‐di‐GMP and EDR proved that TB‐EPS served as important component in the structure and growth of denitrification biofilm, denitrifiers could effectively receive electrons from both acetate and EPS, which was beneficial to maintain relatively high denitrification capability at low temperature. *Pseudomonas* played an important role to maintain stable structure by EPS production under shock organic loading rate and low temperature conditions, and the specialized communities suggested that good nitrate removal property could be enhanced not only by the interaction of unique OTUs but also through the high bioactivity of dominant species.

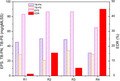

## Introduction

The new standard issued for wastewater discharge in China is stringent, and total nitrogen (TN) is the most difficult index for wastewater treatment plants (WWTPs). TN removal through conventional activated sludge (CAS) process is significantly influenced by operational conditions, including the lack of influent carbon source content, inhibition of refractory organic compounds from industrial wastewater and low temperature (Welander and Mattiasson, 2003; Zhang et al., 2016a, 2016b,2016a, 2016b; Zhao *et al*., 2018). Therefore, the application of denitrification suspended carriers is principally adopted by WWTPs, and prior to conventional activated sludge process, denitrification biofilm presented outstanding performances exposed to shock organic loading rate, and low temperature conditions (Welander and Mattiasson, 2003; Wang *et al*., 2019). However, the TN removal efficiency of denitrification biofilm is merely comparable to CAS process (Zhang et al., 2016a, 2016b,2016a, 2016b), and the possible reason could be due to the existence of extracellular polymeric substance (EPS; Sheng *et al*., 2010).

Previous studies on the use of suspended carriers investigated the biofilm structure owing to the distribution of protein and polysaccharides from EPS and the attachment/detachment of microorganisms on the biofilm (Adav *et al*., 2008; Sheng *et al*., 2010). The secretion of EPS sharply decreases under adverse operation conditions, leading to poor cell adhesion and biomass aggregation (Sheng *et al*., 2010; Tan *et al*., 2014). Protein and polysaccharides are the main substances responsible for biofilm formation (McSwain *et al*., 2005; Adav *et al*., 2008). These substances maintain a stable structure through binding strength and serve as the energy source for biomass growth and propagation (Wang *et al*., 2008; Ye *et al*., 2011). EPS signifies as the energy source to maintain the microbial activity when carbon source was scant (Wang *et al*., 2008; Ye *et al*., 2011); moreover, the resistance of toxic substances derived from influent and temperature gradient can be gradually formed in biofilm due to the compact structure of EPS (Sheng *et al*., 2010; Wang *et al*., 2012). The microbial community in biofilm will undergo obvious structural variation under adverse conditions, and stable microbial community structure could gradually form as the process time extended. Although the discrepancy of EPS was observed in the responses of microbial community under adverse conditions (Sheng *et al*., 2010; Wang *et al*., 2017a), the response mechanism and variation of EPS were not investigated in detail, and the further correlation of EPS components and microbial community structure was still scant.

Cells or strains transfer information through low‐molecular‐weight molecules (Wang *et al*., 2017a) and subsequently enhance cell signal transduction. Cyclic diguanylate (c‐di‐GMP) molecules provide information in accordance with polysaccharide biosynthesis, which is beneficial to adhere to suspended cells in biofilm formation (Zhang *et al*., 2011; Wan *et al*., 2013). C‐di‐GMP is an important intracellular regulator of biofilm structure stabilization and polysaccharide production, and extracellular protein has a relatively high level of c‐di‐GMP (Simm *et al*., 2004; Thormann *et al*., 2006; Merritt *et al*., 2010). However, detailed research on the variation mechanism of EPS and the correlation of EPS and microbial community under adverse operation conditions remains lacking. C‐di‐GMP presents remarkable fluctuation under adverse operation conditions (Wan *et al*., 2013; Wang *et al*., 2017a); therefore, the relevance between c‐di‐GMP transduction and EPS production is beneficial to understand the function of EPS in denitrification biofilm.

Denitrification process was highly associated with electron transfer (Wan *et al*., 2019), and the extracellular protein was considered as the transient media for microbial electron transfer for the good conductive property (Xiao *et al*., 2017); thus, the investigation of EPS function based on electron equilibrium provided new insight into the maintenance of biofilm structure and TN removal capacity. In addition, with the development of high‐throughput sequencing and metagenomics, predominant bacteria were analysed in depth, and the microbial functions of bacterial genera were investigated extensively (Wang *et al*., 2019). Therefore, detailed research on the vital bacterial strains involved in EPS biosynthesis and c‐di‐GMP regulation can be identified, which facilitates to highlight the superiority of denitrification biofilm under adverse conditions.

In order to deeply analyse the adaptability of denitrification exposed to adverse conditions, this study investigated the intracellular c‐di‐GMP and the calculation of electron equilibrium in denitrification process, aimed to discover the function of EPS, and the correlation of EPS and microbial community structure was further discussed. Accordingly, the responses of EPS and microbial community under low organic loading rate, shock organic loading rate and low temperature conditions were explored in detail. The microbial community structure was characterized to identify the dominant and functional genera under adverse conditions, which could enhance the interaction of EPS and c‐di‐GMP during total nitrogen removal process. Finally, this study offers valuable information to elaborate on the total nitrogen removal mechanism of denitrification biofilm, and further provide possible regulation methods for high total nitrogen removal under adverse conditions.

## Results and discussion

### Basic characteristics of denitrification suspended carriers

R2, R3 and R4 showed a slight decrease in biomass during the initial inoculation period of the denitrification suspended carriers, and biofilm debris was observed in R2 and R4 (Fig. S1). This result suggested that the low organic loading rate and low temperature significantly influenced the bioreactor performance. However, the MLSS and MLVSS gradually increased along with the operation of bioreactors and remained stable at day 30 (Table 1). The MLVSS contents of the denitrification suspended carriers in R1 to R4 were 6.27 ± 0.15, 6.58 ± 0.15, 7.03 ± 0.15 and 7.57 ± 0.18 g m^−2^ respectively. The biomass sharply increased in the slope of R4, signifying the remarkable adaptation of the denitrification suspended carriers to low temperature through community succession (Liu *et al*., 2015). Nevertheless, compared with the biomass in R1, the biomass of the denitrification suspended carriers in R2 only increased by a factor of 4.9% due to the fast‐famine condition that inhibited biofilm growth. The relatively high biomass in R3 and R4 indicated that the adverse operational conditions (shock organic loading rate and low temperature) exerted limited effect on biofilm growth, which implied that the applicability of the denitrification biofilm favoured nitrate removal than activated sludge (Seetha *et al*., 2010; Figdore *et al*., 2018).

**Table 1:** Characteristics of denitrification suspended carriers under different application strategies.

	R1	R2	R3	R4
MLSS (g m^−2^)	12.85 ± 1.27	13.43 ± 1.38	12.91 ± 1.29	12.70 ± 1.33
MLVSS (g m^−2^)	6.27 ± 0.15	6.58 ± 0.15	7.03 ± 0.15	7.57 ± 0.18
MLVSS/MLSS (%)	0.49	0.49	0.54	0.60
*ρ* (g cm^−3^)	0.031	0.030	0.030	0.031
*L* (μm)	202	180	205	210

### Nitrate conversion and removal of denitrification suspended carriers

#### Nitrate conversion

**Fig. 1:**
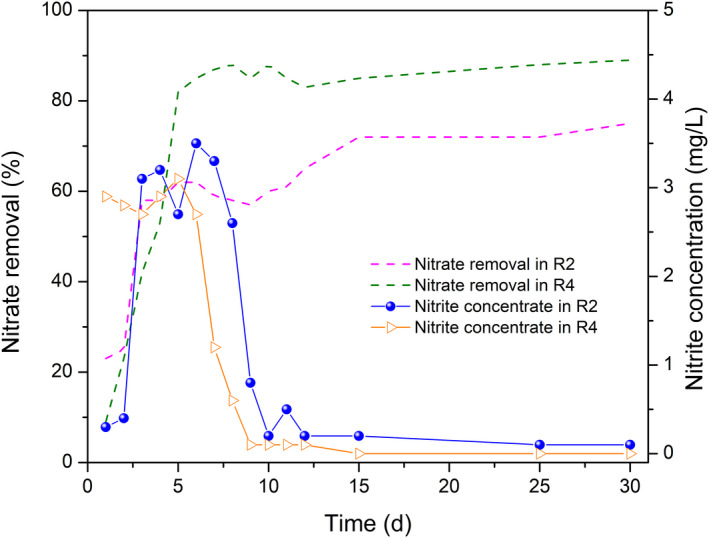
Variation of nitrate removal and nitrite concentrations from R2 and R4.

The nitrate reduction was above 90% in 3 days from R1 and R3 (Fig. S2). Nitrate removal and effluent nitrate content were not influenced by the shock organic loading rate, which indicated that the synthesis and consumption of polysaccharide (Wan *et al*., 2016) from TB‐EPS were alternate along with the operation of R3, thereby improving the denitrification process. The denitrification biofilm from R2 showed poor performance in nitrate removal and nitrite accumulation for 10 days (Fig. 1). Due to the low organic loading rate, the denitrification biofilm was subcultured in the endogenous respiration phase, which resulted in the preferential utilization of the carbon source for biomass growth. By using activated sludge to enhance nitrate removal at low organic loading rate, Shin and Nam (2000) found that the denitrification efficiency was enhanced up to 63%, but the average nitrate reduction from R2 was 71.0%, which implied that the denitrification biofilm was favourable for nitrate removal. Due to the nitrate respiration out‐competing nitrite respiration for limited acetate electrons (Oh and Silverstein, 1999), the nitrite content accumulated to 3.5 mg l^−1^ from R2 during denitrification. With similar numerical values in nitrite production and nitrite consumption (Si *et al*., 2018), nitrite accumulation was finally eliminated. The nitrate removal was approximately 87.9%, suggesting that the denitrification biofilm adapted to the low temperature condition. The nitrite accumulation was observed in R4, indicating that the repression of nitrite‐oxidizing bacteria under low temperature was more serious than that of ammonium‐oxidizing bacteria, and the low rate of nitrite reduction led to nitrite accumulation (Wu *et al*., 2016).

### Nitrate removal

**Fig. 2:**
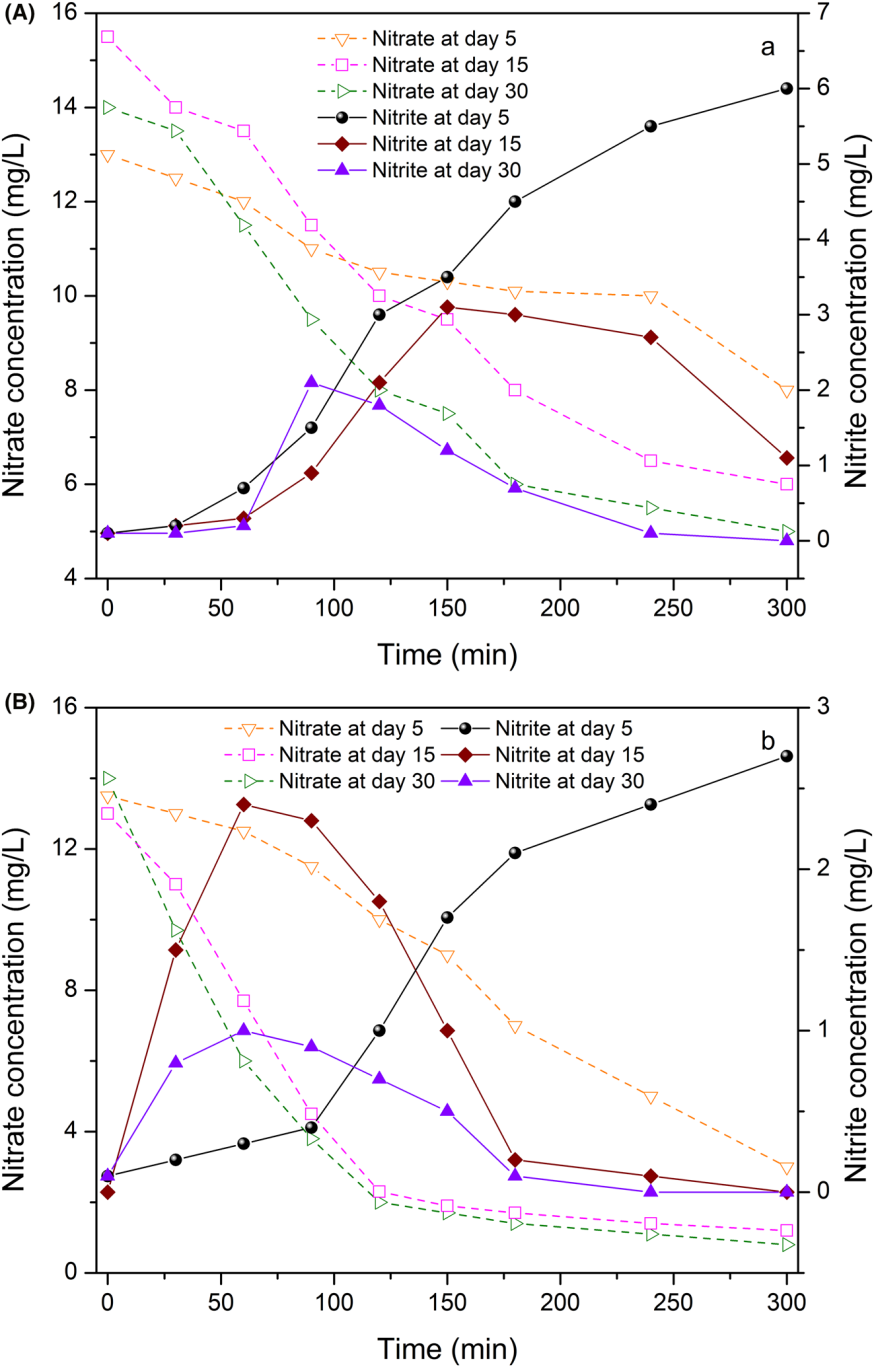
Variation of nitrate and nitrite concentrations from R2 and R4. A. Bioreactor R2. B. Bioreactor R4.

Nitrate removal significantly increased along with the operation of R1 and R3, and the denitrification rates of the denitrification suspended carriers were 5.77 and 6.10 gNO_3_^−^‐N m^−2^·d^−1^. This result indicated that the denitrification suspended carriers can enhance denitrification under shock organic loading rate. The denitrification capacity of the suspended carriers in R2 is shown in Figure 2A, and the nitrate content decreased from 14.0 mg l^−1^ to 5.1 mg l^−1^ with a denitrification rate of 5.10 gNO_3_^—^N m^−2^·d^−1^ (day 30). The denitrification rate decreased by a factor of 0.12, indicating that the influent carbon source and polysaccharides in EPS were primarily utilized for biomass growth (Laurin *et al*., 2006) and thereby led to the relatively poor denitrification performance. However, 240 min of denitrification phase was required to gradually remove the nitrite accumulation, which did not favour nitrate removal. The denitrification suspended carriers obtained a stable operation after 15 days (Fig. 2B) because the effluent nitrite and nitrate contents were comparatively low, and the nitrate content sharply decreased in the slope, thus inducing the good denitrification property of the suspended carriers under low temperature. The denitrification rate in R4 was 5.90 gNO_3_^−^‐N/m^−2^·d^−1^ (day 30), which was slightly higher than that in R1. This result could be attributed to the formation of a temperature gradient by EPS in the denitrification biofilm (Adav *et al*., 2008). The experimental results above showed that the denitrification suspended carriers with less footprint performed good capability in nitrate removal. The application of the denitrification suspended carriers was the greatest in shock organic loading rate, then in low temperature, and lastly in low organic loading rate.

### EPS variation of denitrification suspended carriers

#### Transformation of EPS

**Fig. 3:**
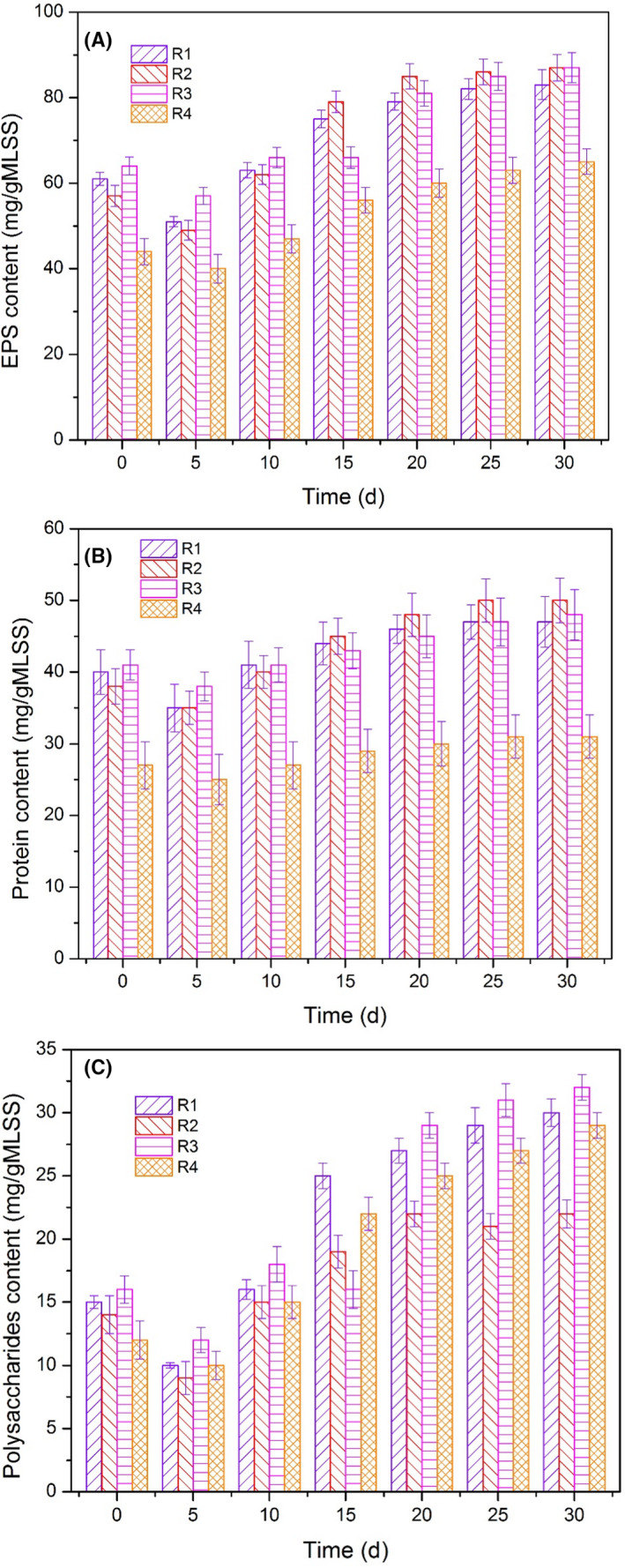
Variation of EPS and TB‐EPS contents from R1 to R4. A. EPS content. B. Protein content in TB‐EPS. C. Polysaccharides content in TB‐EPS.

EPS are microorganism‐secreted metabolic products that accumulate on the surface of bacterial cells (Adav *et al*., 2008). The complex polymers mainly consisting of protein (PN) and polysaccharides (PS) play an important role in altering the physico‐chemical characteristics of cellular surface (Liu *et al*., 2004; Sheng *et al*., 2010). As shown in Figure 3A, the EPS content decreased in the first 5 days due to the poor adaptability of the denitrification suspended carriers to the adverse operational conditions. The biodegradation of EPS under adverse operational conditions (Wang *et al*., 2008) is generally ubiquitous, and the maintenance of microbial activity mainly depends on the energy source provided by EPS (Ruijssenaars *et al*., 2000; Ye *et al*., 2011). The EPS contents progressively increased and remained stable along with the operation of bioreactors, which facilitated cell adhesion and biofilm formation, and enhanced denitrification.

The TB‐EPS content was significantly higher than the LB‐EPS content, as shown in Figure 3B and C, and Figure S3a and b. The TB‐EPS was identified as the main factor in biological adhesion and biomass aggregation. The average biosynthesis rate of polysaccharides was 0.55 ± 0.05 mg g^−1^ MLSS·d in R1, which was comparatively higher than that of protein (Fig. 3B and C). Sheng *et al*. (2010) and Zhu *et al*. (2015) observed that PN maintains the stability of the interior structure and the biomass retention ability of the biofilm based on the high binding strength, and PS further improves microbial adhesion and maintains the stability of the exterior structure. The denitrification suspended carriers were directly transferred to the bioreactors, indicating that the interior structure of the biofilm could be maintained. The biomass decreased in the first 5 days of operation (Fig. S1) demonstrated that the bacterial adhesion capacity was reduced and led to biofilm deterioration and exterior structure demolition. Consequently, the increase in PS was regarded as the key component in biomass aggregation and biofilm formation. The LB‐EPS contents decreased (Fig. S2a and b) along with the increase in TB‐EPS content, indicating that the transformation from LB‐EPS to TB‐EPS was beneficial to maintain the stable structure of the denitrification suspended carriers.

#### Components conversion of EPS

Due to the shock organic loading rate, the PS content at day 15 in R3 decreased to 16.1 mg g^−1^ MLSS, whereas the PN content still increased, indicating that PS served as the energy source for biofilm growth (Durmaz and Sanin, 2001) and nitrate reduction. Similarly, the PS content in R2 was remarkably lower than that in R1, implying that the lack of carbon source resulted in slow biomass growth and PS was utilized as the substrate for bioactivity maintenance. However, the PN content in R2 was higher than that in R1 after 15 consecutive days of operation due to a relatively low protein degradation rate (Vu *et al*., 2009). Moreover, the PN/PS ratio was higher in R2 than in R1, R3 and R4, which implied that the stabilization of the denitrification biofilms in R2 was enhanced by the adhesion of cells through a polymeric matrix (Adav *et al*., 2008; Wang *et al*., 2017b). The PS and PN contents from TB‐EPS in R4 were significantly lower than that in R1 because the biosynthesis rate was severely inhibited at low temperature. The PS content in R4 decreased to 9.9 mg g^−1^ MLSS in the first 5 days, the PS content increased progressively, and the corresponding content reached its maximum of 29.7 mg g^−1^ MLSS. Nevertheless, the PN content in R4 increased only by a factor of 0.15, suggesting that the stable PN content built a cross‐linked network to maintain internal biofilm structure (McSwain *et al*., 2005; Tan *et al*., 2014) and led to the rapid growth of PS (Sheng *et al*., 2010; Limoli *et al*., 2015; Zhang *et al*., 2015), which could further enhance microbial adhesion and improve nitrate removal.

### c‐di‐GMP of denitrification suspended carriers

#### Variation of c‐di‐GMP

**Fig. 4:**
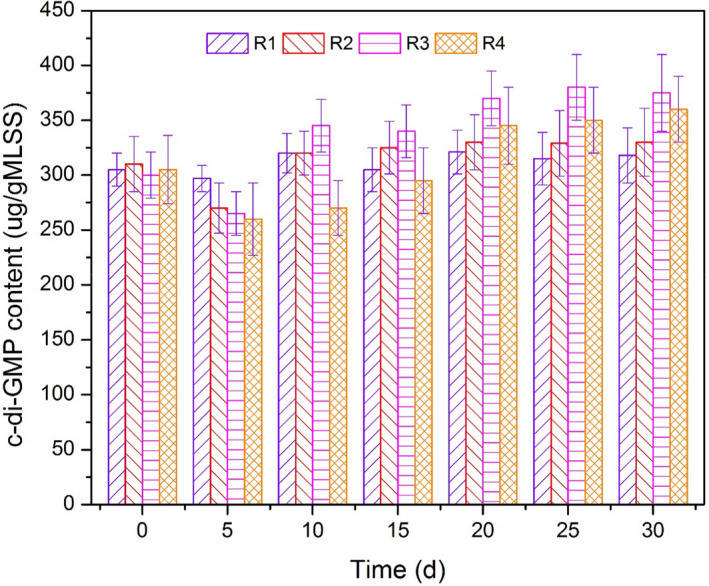
Variation of c‐di‐GMP contents from R1 to R4.

C‐di‐GMP presented as a vital signal messenger to the stability of the biofilm structure, as shown in Figure 4. The c‐di‐GMP content decreased in the first 5 days of operation, which was consistent with the detachment of biomass in R1 to R4. The release of c‐di‐GMP was stable from 305 µg g^−1^ to 318 µg g^−1^ MLSS along with the operation of R1 because the influent quality was constant. In R2, the c‐di‐GMP content decreased to 271.9 µg g^−1^ MLSS at day 5 and then gradually increased to 326.8 µg g^−1^ MLSS, which was slightly higher than that in R1 due to polysaccharide biosynthesis (Whiteley and Lee, 2015). The PS content in TB‐EPS from R2 was lower than that in R1 (Fig. 3C), and the denitrification biofilm in R2 required more energy source to maintain the bioactivity. Thus, the c‐di‐GMP content sustained a high level to produce sufficient polysaccharides (Colvin *et al*., 2012) for denitrification. The c‐di‐GMP content increased up to 375.8 µg g^−1^ MLSS from R3, which indicated that the selective pressure (Wang *et al*., 2017a) on the basis of shock organic loading rate promoted polysaccharide secretion and subsequently enhanced suspended cell adhesion (Wan *et al*., 2013). Low temperature is the rate‐limiting step for polysaccharide biosynthesis (Hu *et al*., 2017), but the PS production in TB‐EPS (R4) drastically increased from 14.9 mg g^−1^ to 29.7 mg g^−1^ MLSS due to the good cell signal transduction by c‐di‐GMP. The c‐di‐GMP content in R4 increased to as high as 360.0 µg g^−1^ MLSS, which implied that the dynamic succession of psychrophiles and psychrotrophs retained microbial activity and released sufficient c‐di‐GMP (Hendrickx *et al*., 2012; Whiteley and Lee, 2015). This phenomenon enhanced the stable structure and nitrate reduction of the denitrification biofilm. In addition, the protein amplification in TB‐EPS was not apparent to the increase in c‐di‐GMP content, but the production of protein was regulated by c‐di‐GMP (Ahimou *et al*., 2007). This result suggested that c‐di‐GMP played an important role in biomass retention and biofilm formation.

#### Correlation of c‐di‐GMP and EPS

The correlation analysis of c‐di‐GMP and microbial characteristics are listed in Table 2. The polysaccharide contents in TB‐EPS and EPS presented a significant positive correlation with c‐di‐GMP content. EPS is a complex mixture of polymers, and their main component TB‐EPS maintains the structure of the denitrification biofilm (Sheng *et al*., 2010; Liu *et al*, 2018). The relatively high content of c‐di‐GMP regulated and promoted the secretion of EPS and the production of polysaccharides (Whiteley and Lee, 2015), which subsequently enhanced the microbial adhesion and biofilm stabilization (Schirmer, 2016). Polysaccharides were utilized as the energy source, being favourable for the denitrification process, particularly under low organic loading rate and low temperature conditions (Fig. 3A and C). Moreover, the protein content in TB‐EPS was positively correlated with c‐di‐GMP content. The biosynthesis of hydrophobic groups (Cao *et al*., 2016) from protein was modulated by c‐di‐GMP through genes encoding for enzymes (Kumar *et al*., 2018). Therefore, the increase in c‐di‐GMP content was conducive to biomass aggregation, which improved the stabilization of the denitrification biofilms. In addition, a positive correlation was established between c‐di‐GMP and the biomass (MLVSS) and thickness (L) of the biofilm. The increase in biomass and thickness induced a high EPS production and formed a complete anoxic condition to maintain denitrifiers and enhance nitrate removal (Adav *et al*, 2008; Pronk *et al*., 2015).

**Table 2:** Correlation analysis of c‐di‐GMP and microbial characteristics.

	c‐di‐GMP	
	*r*	*P*
MLVSS (g m^−2^)	0.732	0.004
*ρ* (g cm^−3^)	0.659	0.005
*L* (μm)	0.765	0.004
EPS content (mg g^−1^MLSS)	0.905	0.002
TB‐EPS content (mg g^−1^MLSS)	0.901	0.002
Protein content in TB‐EPS (mg g^−1^MLSS)	0.825	0.004
Polysaccharide content in TB‐EPS (mg g^−1^MLSS)	0.910	0.001

#### Electron equilibrium

**Fig. 5:**
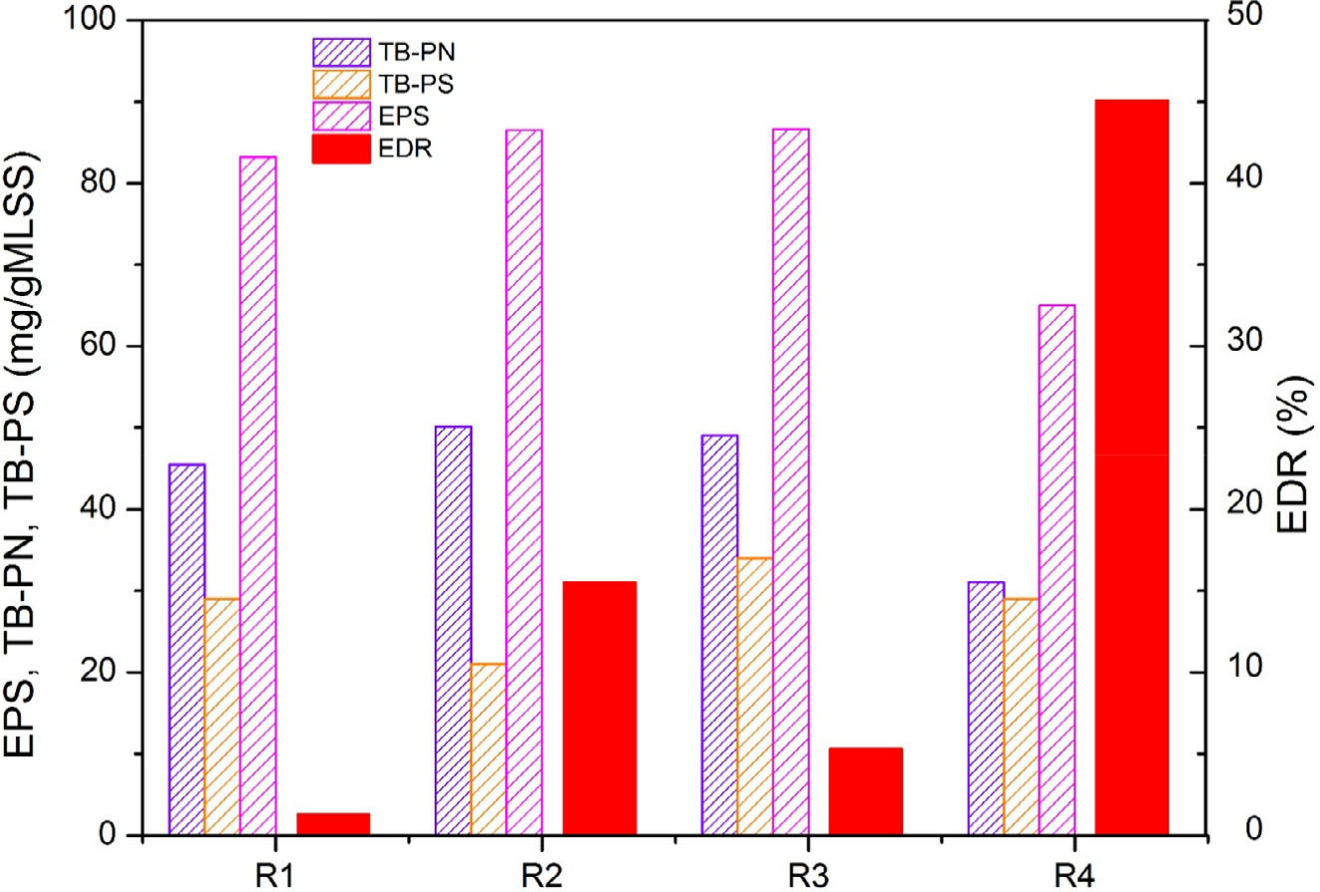
Electronic deletion of bioreactors in steady state.

As depicted in Figure 5, it was conspicuous that the electron deletion (Chen *et al*., 2010) was universal under adverse conditions. Additional carbon source was utilized except acetate with an electronic deletion rate peak at 45.1% in low temperature condition. The EPS content was the lowest in R4, indicated that EPS was employed as the carbon source to maintain the growth rate and nitrate reduction capacity by denitrification biofilm. Remarkable negative correlation between EDR and TB‐PN and TB‐PS was discovered (Fig. 5), implying that a huge amount of TB‐PN and TB‐PS were utilized; however, the ratio of TB‐PN and TB‐PS (TB‐PN/TB‐PS) was well maintained, suggested the biofilm structure was stable (Sheng *et al*., 2010) in R4, which was conducive to provide good denitrification circumstance at low temperature. In addition, Figure 5 displays that the EDR, EPS, TB‐PN and TB‐PS in R3 have a remarkably positive correlation with those in R1 (*R* ^2^ = 0.9998), although EPS was similarly oxidized as that in R4, shock loading rate possessed little impact to the stability and denitrification capacity of biofilm. EDR in R2 was relatively higher than that in R1, while the TB‐PS content was lower, indicated that TB‐PS was utilized as carbon source to maintain the growth of microorganism (Wang *et al*., 2008; Ye *et al*., 2011). However, the TB‐PN content was close to that in R1, denoted that comparatively high TB‐PN/TB‐PS decrease the stability of biofilm and further resulted in the inhibition of denitrification bioactivity of biofilm (Sheng *et al*., 2010). In addition, extracellular electron transfer was enhanced based on the good nitrate reduction performance and relatively stable content of TB‐PN (Liu *et al*., 2014; Xiao *et al*., 2017) in R4 (Fig. 3B); thus, denitrifiers could effectively receive electrons from acetate and EPS, which was beneficial to maintain relatively high denitrification capability at low temperature.

### Microbial community structure

#### Richness and diversity of microbial community

The richness and diversity are listed in Table S1. The richness in accordance with the Chao and ACE values in R1 showed that the microbial community was abundant. The richness in accordance with the Chao and ACE values in R1 showed that the microbial community was abundant, and the richness of microbial community increased along with the operation of the bioreactor at low temperature (R4); however, the richness of R2 and R3 presented a decreasing trend. The Chao and ACE indices in R1 were relatively higher than those in R3 and R4 but relatively lower than those in R2. The richness of microbial communities decreased under shock organic loading rate and low temperature conditions but increased under low organic loading rate conditions, indicating that the carbon source content was the rate‐limiting step for denitrification (Spinelli *et al*., 2018) and that high microbial diversity promoted nitrate removal.

Based on the Shannon and Simpson indices, the diversity of microbial community in R2 was the highest at day 15 (Table S1), but the nitrate removal was relatively low, inducing that the low carbon source significantly influenced denitrification. Nevertheless, the diversity in R4 was higher than that in R2 at day 30, indicating that the microbial diversity was richer under low temperature than under low organic loading rate, and the denitrifiers could progressively adapt to adverse conditions and achieve good nitrate removal performance. Furthermore, no overall change in microbial diversity was observed in R3, and the Shannon and Simpson values were obviously lower than those found under superior carbon source and low oxygen conditions (Shen *et al*., 2013; Liu et al., 2017a, 2017b,2017a, 2017b). This result implied that the shock organic loading rate rarely influences the microbial structure of denitrifiers and conversely reinforced the dominant microbial populations.

### Relationship among microbial communities

**Fig. 6:**
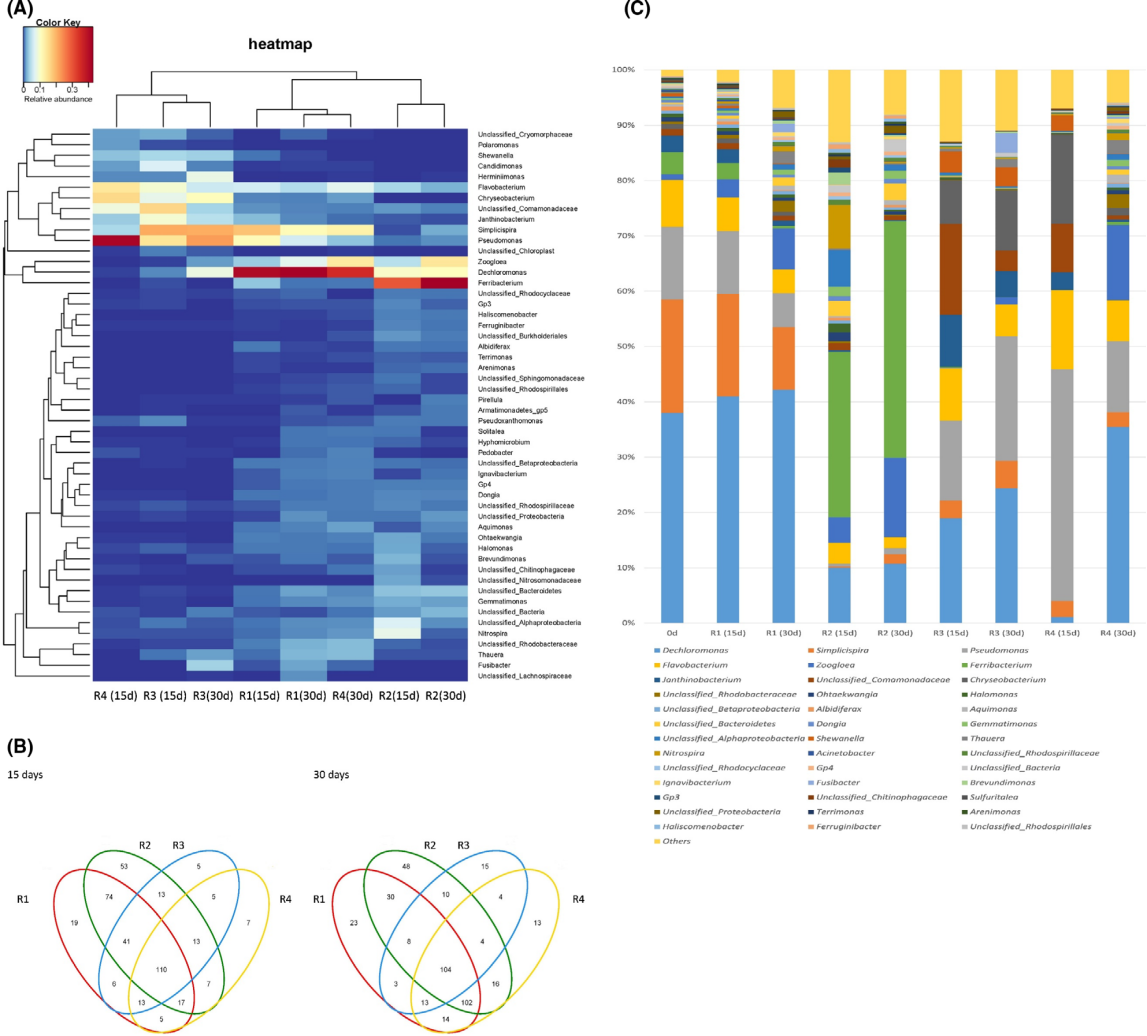
Structure of microbial community at the genus level. A. Hierarchically clustered heat map. B. Venn diagram. C. Microbial community structure.

A hierarchically clustered heat map was depicted to present the overall view of the identified interactions among R1 to R4 (Fig. 6A). Slight shifts of microbial communities were observed for R1, R2 and R3, while excess shifts were obvious for R4. The heat map revealed that the microbial community in R2 showed higher similarity with that in R1, and the microbial community in R3 was markedly different from that in R1. However, surprisingly dynamic microbial community was noted in R4, which dramatical differences were found at day 15 and the similarity of microbial community was close to that in R1 at day 30. The possible reason could be due to the drastic community succession under low temperature conditions (Hoang *et al*., 2014), and the microbial activity gradually recovered along with the operation of R4. In addition, with the relatively high nitrate removal performance, the similarity of microbial community in R3 and R4 was greatly different at day 30, implying there could be discrepancy between the dominant species for denitrification.

The venn diagram based on common and unique operational taxonomic unit (OTU) was employed to evaluate the difference and similarity among microbial communities (Fig. 6B). The total observed OTUs of R1 to R4 changed from 285, 328, 206 and 177 to 297, 322, 161 and 270 respectively. The obvious OTUs variation was discovered in R3 and R4, in which 45 OTUs were eliminated in R3 while 93 OTUs were enhanced in R4. The relatively high nitrate removal was attributable to the dominant species in R3, whereas it was owing to the microbial diversity and synergistic effect in R4, which was consistent with the ACE and Chao indices of microbial community listed in Table S1. 110 OTUs were shared by R1 to R4 at day 15 and 104 OTUs were shared by R1 to R4 at day 30, indicating the unique OTUs covered the majority of total observed OTUs. The number of specialized communities in R1 to R4 at day 15 was 19, 53, 5 and 7 respectively. However, 48 OTUs in R2 were detected at day 30, implying that the microbial community progressively adapted to low organic loading rate conditions and subsequently produced polysaccharides as energy source (Wang *et al*., 2008) for denitrification.

The specialized communities in R3 at day 30 notably increased while the total OTUs apparently decreased, moreover, the specialized communities in R4 at day 30 slightly increased whereas the total OTUs evidently increased, these findings suggest that the good nitrate removal property could be enhanced not only by the interaction of unique OTUs (R3) but also through the high bioactivity of dominant species (R4).

### Structure of microbial community at the genus level

The denitrification suspended carriers collected in R1 possessed a high microbial diversity, where *Dechloromonas*, *Simlicispira*, *Pseudomonas*, *Flavobacterium* and *Zoogloea* were the dominant genera (Fig. 6C). The respective abundance of *Dechloromonas* and *Zoogloea* increased from 41.0% and 3.3% to 42.2% and 7.5%, whereas the abundance of *Simlicispira*, *Pseudomonas* and *Flavobacterium* decreased from 18.5%, 11.4% and 6.1% to 11.3%, 6.1% and 4.3% respectively. *Dechloromonas* and *Zoogloea* were considered as the important bacterium species in denitrification, and the enrichment of *Dechloromonas* and *Zoogloea* was beneficial for stable nitrate removal (Shen *et al*., 2013; Lv *et al*., 2014). However, the microbial community structure of the denitrification suspended carriers in R2 drastically shifted. *Dechloromonas*, *Zoogloea* and *Ferribacterium* became prevalent, and *Ferribacterium* was the most abundant genus, responsible for 42.8% of the total bacterial sequences. *Ferribacterium* was an obligate anaerobic bacterial genus with organic acid oxidation capacity, and ferric ion was generally utilized as the electron acceptor (Cummings *et al*., 1999), thereby led to the relatively poor denitrification performance under low organic loading rate.

On the basis of the comparatively low Shannon and Simpson values, *Dechloromonas*, *Simlicispira*, *Pseudomonas*, *Flavobacterium*, *Janthinobacterium*, *Unclassified_Comamonadaceae* and *Chryseobacterium* were the predominant genera in R3. The notable increase in bacterial strains *Simlicispira*, *Pseudomonas* and *Chryseobacterium* was associated with biofilm formation, carbon source utilization and denitrification (Lu *et al*., 2007; Pradyut *et al*., 2014; Dutta *et al*., 2018). *Ferribacterium* nearly went extinct due to the excellent denitrification capability in R3, in which the denitrifiers preferentially utilized carbon source for nitrate removal. In addition, *Fusibacter* with the function of transferring a wide range of carbohydrates to acetate and ethanol existed only in R3 (Fadhlaoui *et al*., 2015), which was conducive to utilization of denitrifiers under shock organic loading rate. The abundance of *Dechloromonas*, *Zoogloea* and *Simplicispira* dramatically enriched in R4. The floc‐forming *Zoogloea* bacterium species facilitated the formation of biofilm (Gao *et al*., 2018) and effectively decreased the impact of low temperature (Wang *et al*., 2012), which was suitable for the growth of *Dechloromonas* and *Simplicispira*, and further resulted in good denitrification capability. The relative abundance of *Pseudomonas*, *Flavobacterium*, *Unclassified_Comamonadaceae* and *Chryseobacterium* decreased along with the operation of R4, and the bacterial genera *Unclassified_Comamonadaceae* and *Chryseobacterium* were almost eliminated by low temperature conditions.

### Microbial community and c‐di‐GMP

With the abundance of *Dechloromonas* in R1 to R4, c‐di‐GMP was discovered to possess a tightly bound capacity with YcgR, a protein conserved in *Dechloromonas* (Ryjenkov *et al*., 2006). This result suggested that the secretion of EPS from the denitrification biofilm was initiated through the downstream signal transduction cascade (Whiteley and Lee, 2015). *Flavobacterium*, *Shewanella*, *Thauera*, *Acinetobacter*, *Unclassified Rhodospirillaceae* and *Unclassified_Rhodocyclaceae* were involved in the biosynthesis of polysaccharides regulated by c‐di‐GMP from R1 to R4 (Zhang and Hendrickson, 2010; Amarasinghe *et al*., 2013; Wan et al., 2013, 2015; Whiteley and Lee, 2015), which markedly facilitated the stabilization of the denitrification biofilm structure. Driven by the selective pressure (Liu and Tay, 2002; Wang *et al*., 2017a), the secretion of EPS improved with the increase in c‐di‐GMP content (Figs 4A and 5). The c‐di‐GMP content in *Pseudomonas* was evaluated and showed that the biofilm formation was considerably enhanced through exopolysaccharide production (Huang *et al*., 2003). This result indicated that *Pseudomonas* played an important role to maintain stable structure by EPS production under adverse conditions in R3 and R4, and relatively high denitrification capacity was subsequently achieved. However, no representative bacterial strains demonstrated a high c‐di‐GMP production capacity in R2 because of the relatively low organic loading rate rather than low secretion of EPS by the denitrification suspended carriers.

## Conclusions

The discrepancy of c‐di‐GMP and EDR proved that TB‐EPS served as important component in the structure and growth of denitrification biofilm, denitrifiers could effectively receive electrons from both acetate and EPS, which was beneficial to maintain relatively high denitrification capability at low temperature. Denitrification biofilm was favourable for nitrate removal than activated sludge at low organic loading rate, and the application of the denitrification suspended carriers was the greatest in shock organic loading rate, then at low temperature, and lastly in low organic loading rate. The lack of carbon source resulted in slow biomass growth and TB‐PS was utilized as the substrate for bioactivity maintenance, but the stable TB‐PN/TB‐PS maintained internal biofilm structure and further enhanced microbial adhesion and nitrate removal. C‐di‐GMP content sustained 326.8 µg g^−1^ MLSS to produce sufficient polysaccharides for denitrification at low organic loading rate. The TB‐PS, TB‐EPS and EPS presented significant positive correlation with c‐di‐GMP content, and the TB‐protein was positively correlated with c‐di‐GMP content in accordance with SPSS. Slight shifts of microbial communities were observed for R1, R2 and R3, while excess shifts were obvious for R4. *Pseudomonas* played an important role to maintain stable structure by EPS production under shock organic loading rate and low temperature conditions, and the specialized communities suggested that good nitrate removal property could be enhanced not only by the interaction of unique OTUs but also through the high bioactivity of dominant species.

## Experimental procedures

### Experimental set‐up and operation

**Fig. 7:**
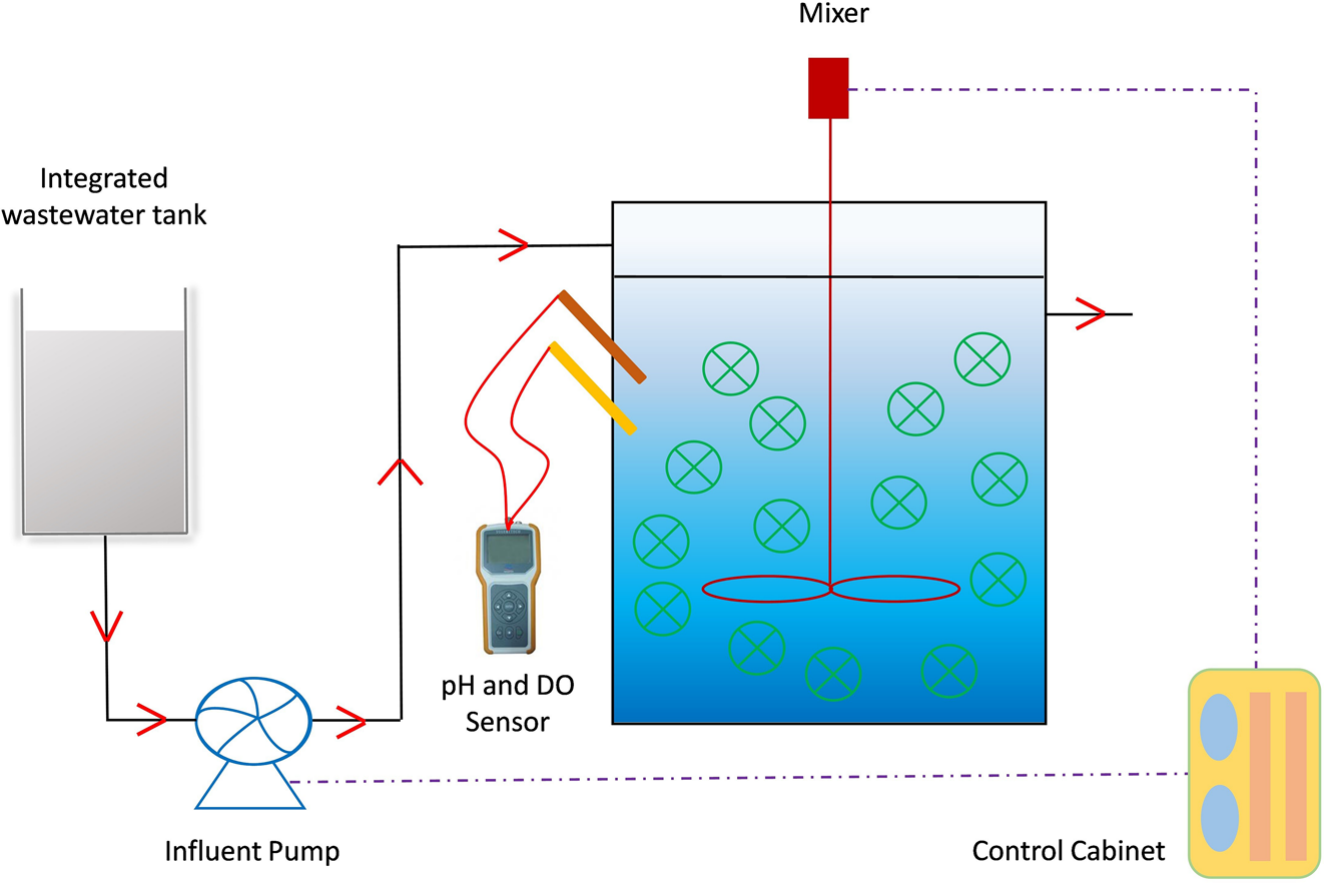
Schematic diagram of denitrification bioreactor.

Denitrification suspended carriers with average mixed liquor volatile suspended solids (MLVSS) of 5500–6000 mg m^−2^ carriers were collected from the anoxic tank in Lucun (Wuxi, China) WWTP through an anaerobic–anoxic‐oxic process. The denitrification suspended carriers were subsequently transferred to bioreactors with a filling ratio of 50%. The applicability of the denitrification suspended carriers was determined in four double‐jacked plexiglass vessels (internal diameter of 24 cm and height of 50 cm, Fig. 7) with an effective volume of 20.0 l. The bioreactors were continuously fed by a peristaltic pump from the feed tank, and the reaction pH, dissolved oxygen (DO) and hydraulic retention time were adjusted to 7.0–7.5, below 0.1 mg l^−1^, and 3 h respectively. Bioreactor R1 was operated as the control group, and bioreactors R2, R3 and R4 were designed to simulate the low organic loading rate, the shock organic loading rate and the low temperature conditions respectively. In addition, the fluctuation of influent in R3 is simulated from the influent characteristics of Lucun (Wuxi, China) WWTP in 2019, and the influent COD is the major variable. The components and concentrations of synthetic wastewater are summarized in Table 3.

**Table 3:** Components and concentrations of synthetic wastewater.

	COD (mg l^−1^)	NH_4_^+^‐N (mg l^−1^)	NO_2_^−^‐N (mg l^−1^)	NO_3_^−^‐N (mg l^−1^)	TP (mg l^−1^)	Temperature (°C)
R1	260–300	0.1–1	0.1–0.3	15–25	3–5	20–25
R2	80–100	0.1–1	0.1–0.3	15–25	3–5	20–25
R3	150–1000	0.1–1	0.1–0.3	15–25	3–5	20–25
R4	260–300	0.1–1	0.1–0.3	15–25	3–5	4–8

### Analytical methods

Chemical oxygen demand (COD), acetate, nitrite and nitrate were regularly measured in accordance with standard methods (APHA, 1998). The biofilm on the surface of the denitrification suspended carriers was generally tightly bounded. Thus, a dropper brush was used to scrape the biomass, and the mixed liquor suspended solids (MLSS) and MLVSS were determined as previously described by Zhang *et al*. (2013). The density (*ρ*) and thickness (*L*) of the biofilms were calculated using the following equations:

(1) $$\rho =\frac{\text{VSS}}{V},$$

(2) $$L=\frac{\text{VSS}}{\rho },$$

where VSS (g m^−2^) represents the biomass of the suspended carriers, and *V* (m^3^ m^−2^) denotes the biofilm volume on the surface area of the carriers (Alves *et al*., 2002; Shi *et al*., 2015). The specific denitrification rates were determined in accordance with the method described by Carvalho *et al*. (2018). DO and pH were monitored using a portable multi‐parameter meter (WTW 3430, Germany).

### Extraction and measurement of EPS

Through the scraping of biomass from the denitrification suspended carriers, EPS extraction was carried out in accordance with the formaldehyde–NaOH protocol (Adav *et al*., 2008). Loosely bound EPS (LB‐EPS) and tightly bound EPS (TB‐EPS) were extracted to explain the evolution of EPS during the applicability of the denitrification suspended carriers. Finally, the total polysaccharides (PS) and total protein (PN) concentrations were quantified by Dubois and Lowry methods, respectively, to evaluate the structure of the denitrification suspended carriers (Lowry *et al*., 1951; Dubois *et al*., 1956).

### Calculation of electron equilibrium

Acetate and nitrate were the electron donator and acceptor in the denitrification process respectively. On the basis of substance transformation and mass balance, the calculations of electron equilibrium were described as the following equations:

(3) $$e1=4e^{‐}\left({\text{Ac}}_{\inf}^{‐}‐{\text{Ac}}_{\text{eff}}^{‐}\right)$$

(4) $$e2=2e^{‐}{\text{nitrite}}_{\text{eff}}+5e^{‐}\left({\text{nitrate}}_{\text{eff}}‐{\text{nitrite}}_{\inf}‐{\text{nitrate}}_{\inf}\right)$$

(5) $$\text{EDR}=\frac{e2‐e1}{e2}\times 100\%$$

where e1 and e2 (mmol l^−1^) represents the loss and reception of electrons in denitrification process; Ac^−^_inf_, nitrite_inf_ and nitrate_inf_ (mmol l^−1^) denotes the influent acetate, nitrite and nitrate contents in each bioreactor; Ac^−^_eff_, nitrite_eff_ and nitrate_eff_ (mmol l^−1^) defiles the effluent acetate, nitrite and nitrate contents in each bioreactor; additionally, EDR (%) expresses the electronic deletion rate in denitrification process.

### Extraction and measurement of c‐di‐GMP

The intracellular second messenger c‐di‐GMP of the denitrification suspended carriers was extracted using the procedures proposed by Wan *et al*. (2013) and subsequently measured through high‐performance liquid chromatography (HPLC; Agilent 1260, Agilent, Santa Clara, CA, USA). Approximately 10 ml of scraped biomass mixture from the denitrification suspended carriers was freeze‐dried at −60°C. Then, 0.2 g of the dried biomass and 15 ml of Milli‐Q water were loaded in 50 ml tubes. The lysozyme was diluted by using a phosphate buffer (pH 7.2) dosed with a terminal concentration of 1 g l^−1^ and then incubated at 37°C for 45 min. After centrifugation at 9000 r.p.m. min^−1^ for 15 min at 4°C, the supernatant was transferred to a new centrifuge tube and diluted with ethanol at ethanol–supernatant ratio of 2. The tube was incubated at 4°C for 1 h with shaking every 5–10 min and then centrifuged at 9000 r.p.m. min^−1^ for 15 min at 4°C. About 3 ml of Milli‐Q water was introduced to dilute the precipitate after incubation at 37°C for 3 h. The mixture was transferred to a new centrifuge tube and then centrifuged at 12 000 r.p.m. min^−1^ for 10 min. Finally, 1 ml of the supernatant was loaded into a chromatogram vial for HPLC analysis. HPLC was performed with a C18 column at 40°C. Detection was conducted at 254 nm using a diode array detector. Runs were performed in a mixed solvent that comprised 95% of Solvent A as acetate–phosphate buffer solution and 5% of Solvent B as 100% acetonitrile at 1 ml min^−1^. Furthermore, correlation analysis with biomass and EPS was expressed using Statistical Product and Service Solutions (SPSS 19.0).

### Microbial community structure

The total DNA of the denitrification suspended carriers was sampled from R1, R2, R3 and R4 at days 15 and 30 using a bacterial genomic DNA extraction kit (ABigen, China). The extracted rDNA was then amplified via PCR using the universal primers 341F (5ʹ‐CCTACGGGNGGCWGCAG‐3ʹ) and 785R (5ʹ‐GACTACHVGGGTATCTAATCC‐3ʹ), which were composed by Takara (Dalian, China). The genomic DNA and PCR products were evaluated by electrophoresis in 1% agarose gels, and positive clones were sequenced on an Illumina MiSeq platform at Genergy Biotechnology (Shanghai, China). Principal component analysis (PCA) was used for genus composition analysis of the denitrification suspended carriers under different operation conditions.

## Conflict of interest

None declared.

## Author contributions

SW involved in project administration; SW and HL involved in roles/writing–original draft; JL involved in writing–review and editing; LZ, SW and QX involved in investigation and methodology. All authors listed have made a substantial, direct and intellectual contribution to the work and approved the final manuscript for publication.

## Supporting information


**Fig. S1.** Variation of MLVSS from R1 to R4.
**Fig. S2.** Variation of nitrate removal and nitrite concentrations from R1 and R3.
**Fig. S3.** Variation of Protein and polysaccharides contents in LB‐EPS from R1 to R4. (a) Protein content; (b) Polysaccharides content.

**Table S1.** Alpha diversity of denitrification suspended carriers under different application strategies
