# Supplementary material for: Correlation of extracellular polymeric substances and microbial community structure in denitrification biofilm exposed to adverse conditions
Source: Microb Biotechnol. 2020 Jul 23;13(6):1889–903. doi: 10.1111/1751-7915.13633 (PMC7533329; doi:10.1111/1751-7915.13633)
Supplement: Supplementary file 1 — Fig. S1. Variation of MLVSS from R1 to R4. Fig. S2. Variation of nitrate removal and nitrite concentrations from R1 and R3. Fig. S3. Variation of Protein and polysaccharides contents in LB‐EPS from R1 to R4. (a) Protein content; (b) Polysaccharides content. [file MBT2-13-1889-s001.docx]

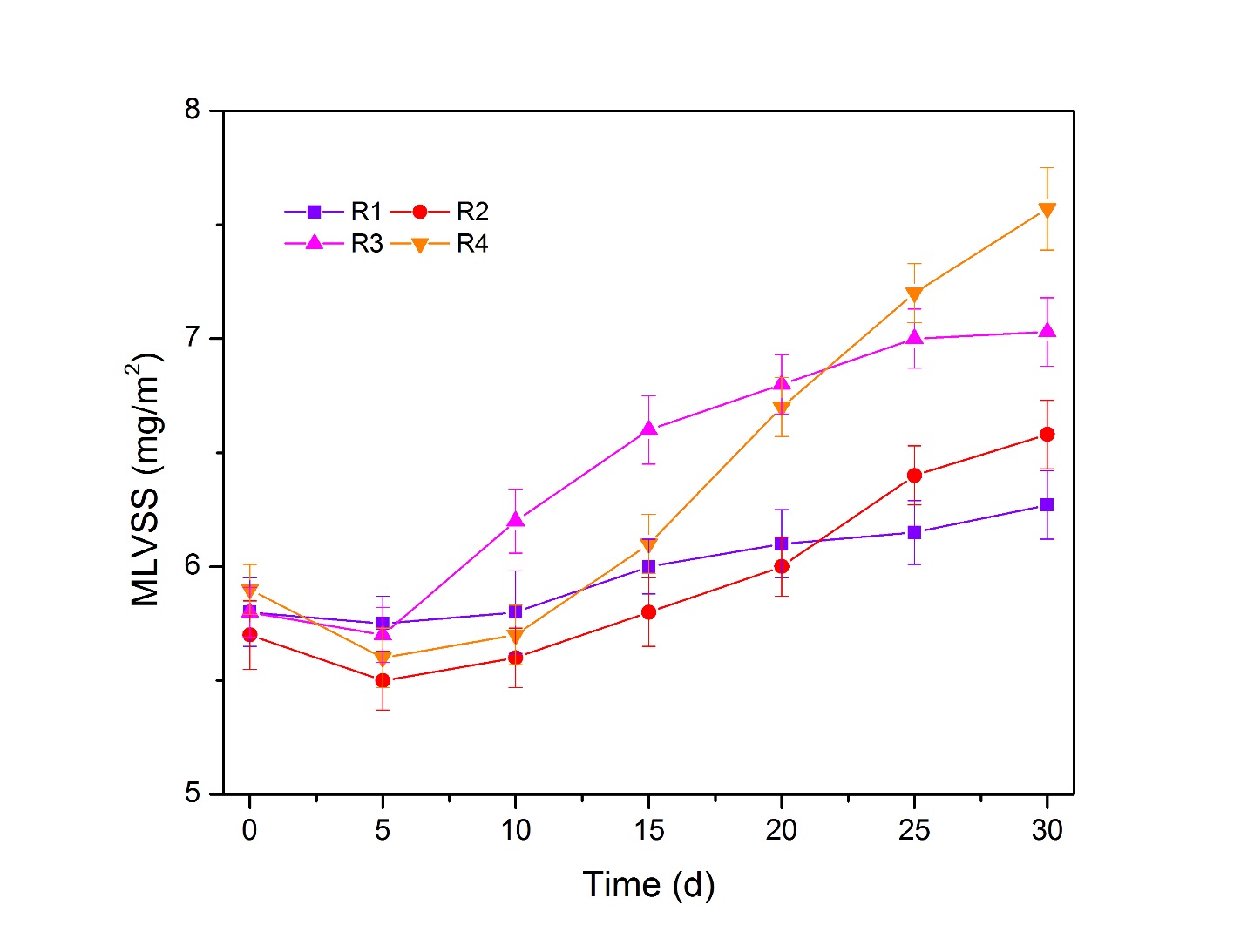
Supplementary Fig. 1 Variation of MLVSS from R1 to R4


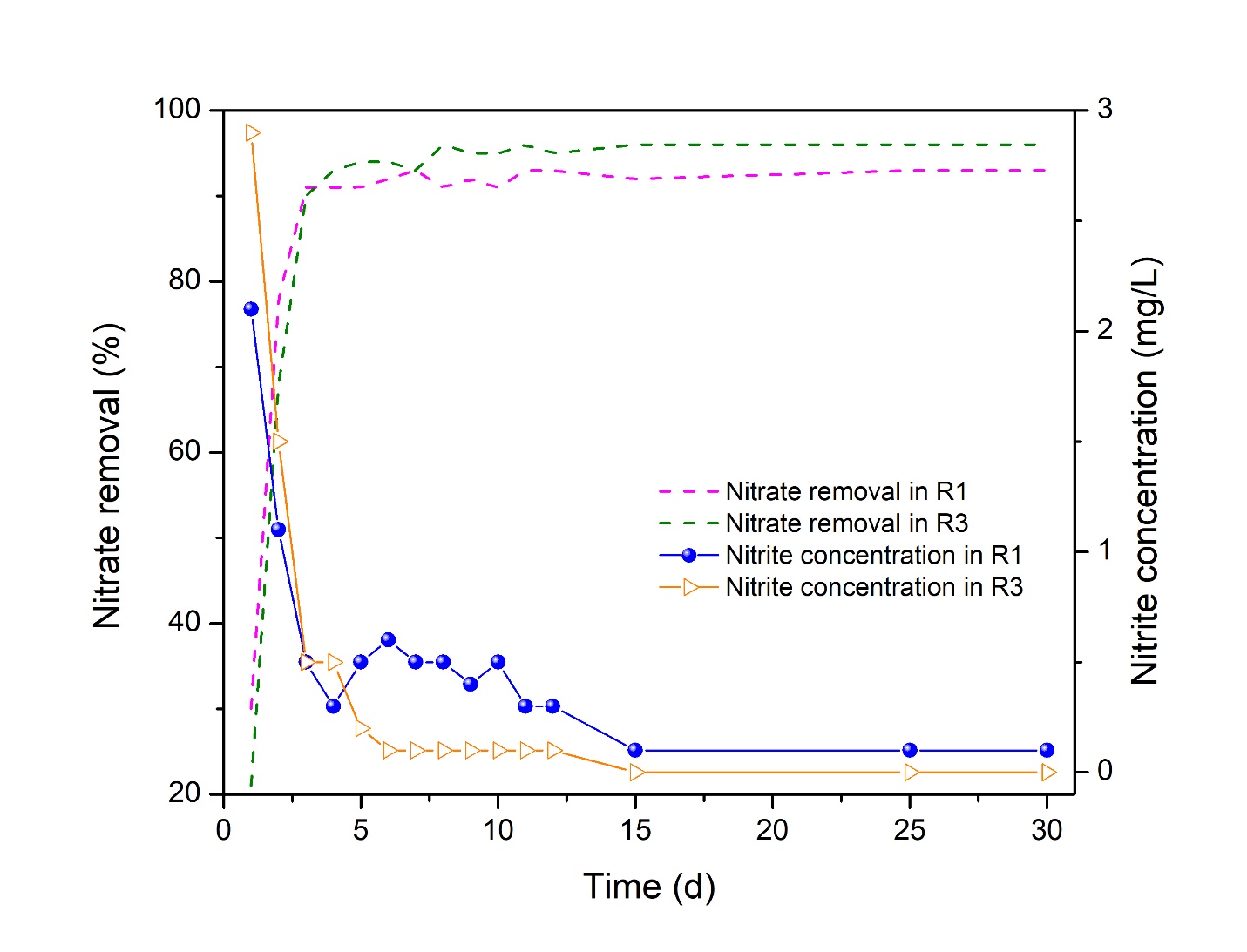


Supplementary Fig. 2 Variation of nitrate removal and nitrite concentrations from R1 and R3


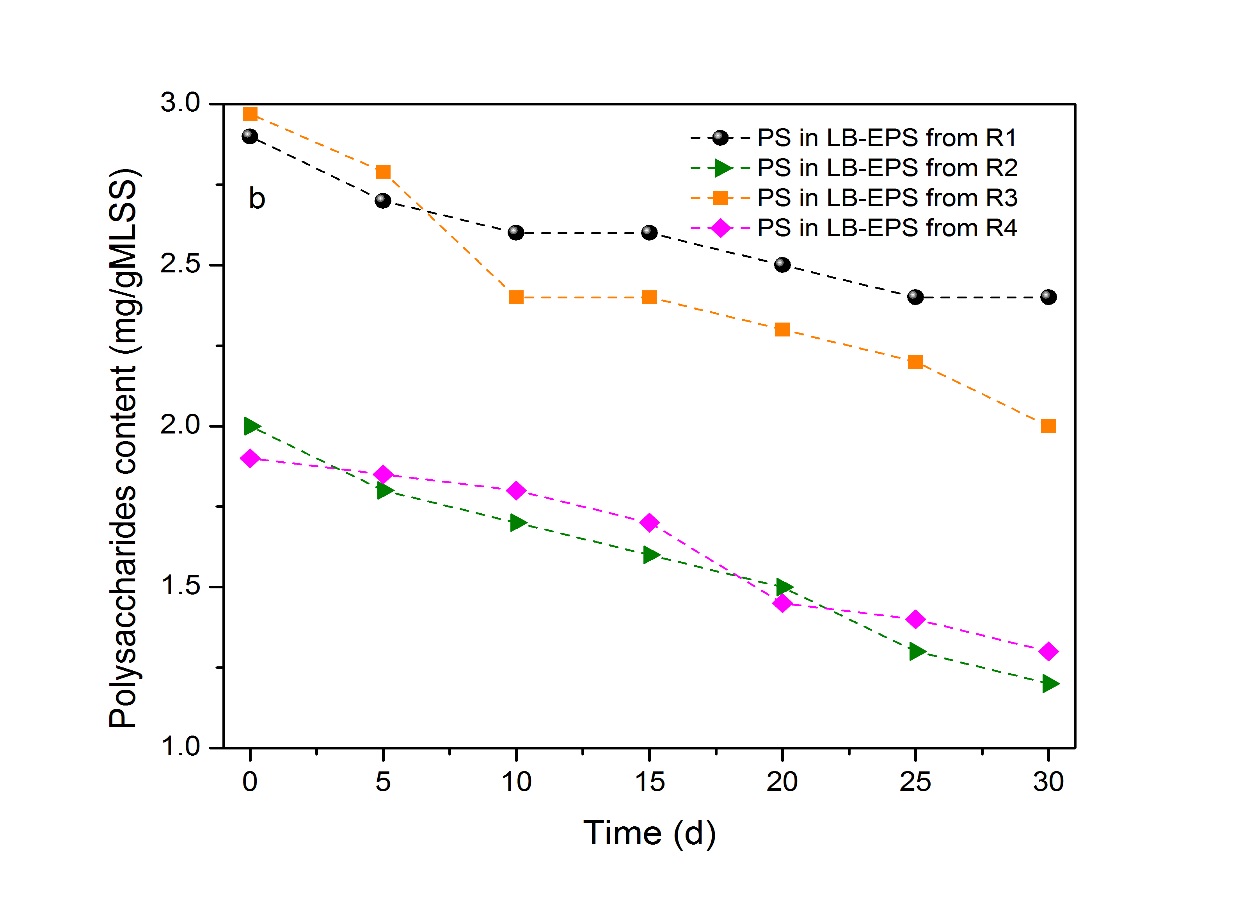

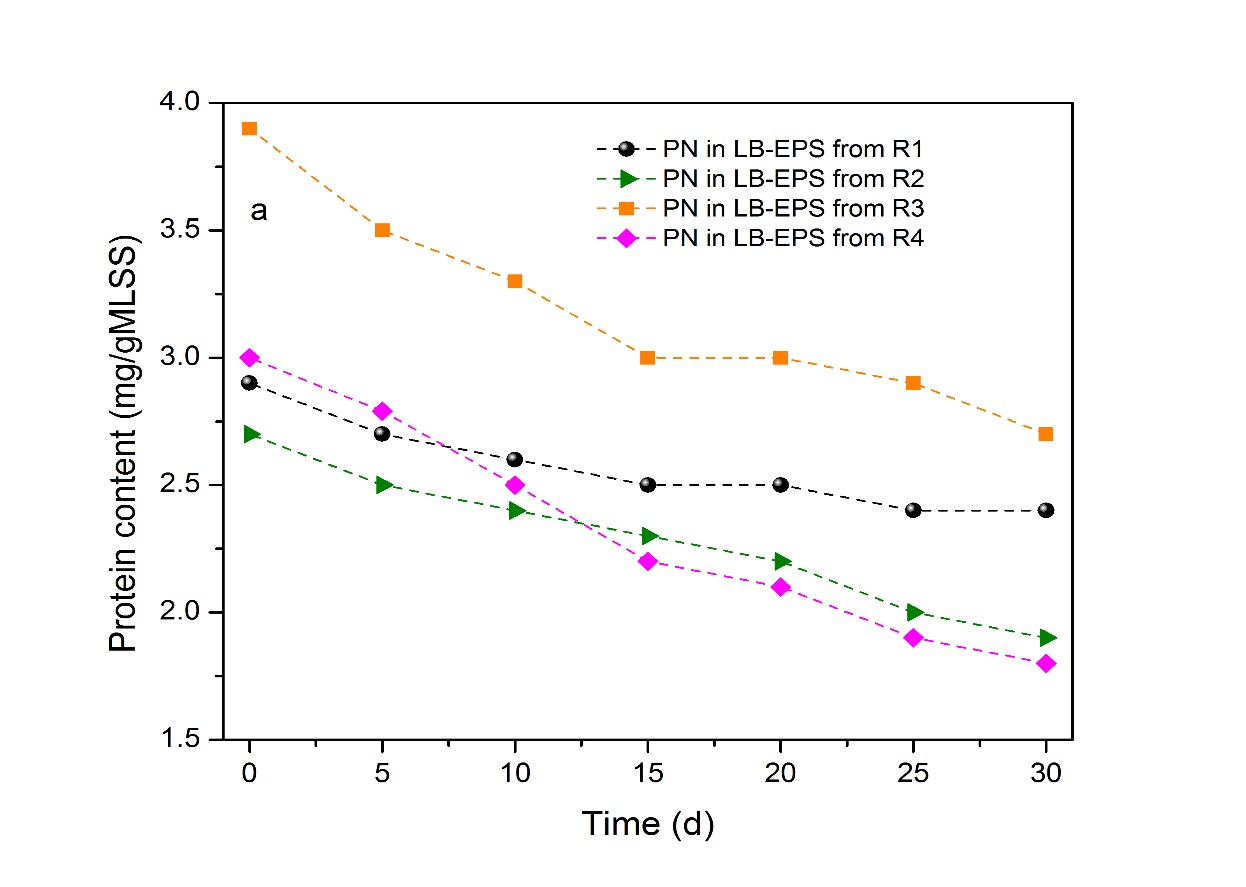
Supplementary Fig. 3 Variation of Protein and polysaccharides contents in LB-EPS from R1 to R4

a) Protein content; b) Polysaccharides content
