# Supplementary material for: Correlation of extracellular polymeric substances and microbial community structure in denitrification biofilm exposed to adverse conditions
Source: Microb Biotechnol. 2020 Jul 23;13(6):1889–903. doi: 10.1111/1751-7915.13633 (PMC7533329; doi:10.1111/1751-7915.13633)
Supplement: Supplementary file 2 — Table S1. Alpha diversity of denitrification suspended carriers under different application strategies [file MBT2-13-1889-s002.docx]

| Supplementary Table 1 Alpha diversity of denitrification suspended carriers under different application strategies | | | | |
| --- | --- | --- | --- | --- |
|  | Ace | Chao | Shannon | Simpson |
| 0 d | 327.015 | 329.075 | 2.501 | 0.149 |
| R1 (15 d) | 332.129 | 331.923 | 2.882 | 0.142 |
| R1 (30 d) | 349.85 | 351.250 | 3.239 | 0.125 |
| R2 (15 d) | 373.519 | 385.000 | 3.673 | 0.105 |
| R2 (30 d) | 355.072 | 356.000 | 3.001 | 0.198 |
| R3 (15 d) | 258.316 | 249.364 | 2.845 | 0.103 |
| R3 (30 d) | 211.839 | 199.607 | 2.827 | 0.095 |
| R4 (15 d) | 254.262 | 244.536 | 2.742 | 0.103 |
| R4 (30 d) | 323.523 | 333.577 | 3.280 | 0.097 |
